# Circulating inflammatory cytokines and sarcopenia-related traits: a mendelian randomization analysis

**DOI:** 10.3389/fmed.2024.1351376

**Published:** 2024-08-13

**Authors:** Aochuan Sun, Saiya Liu, Fen Yin, Zhuangzhuang Li, Zhengtang Liu

**Affiliations:** ^1^Graduate School, Beijing University of Chinese Medicine, Beijing, China; ^2^Xiyuan Hospital, China Academy of Chinese Medical Sciences, Beijing, China; ^3^Dongzhimen Hospital, Beijing University of Chinese Medicine, Beijing, China

**Keywords:** sarcopenia, hand grip strength, appendicular lean mass, usual walking pace, inflammatory cytokines, mendelian randomization analysis

## Abstract

**Objective:**

To explore the causal relationships between 91 circulating inflammatory cytokines and sarcopenia-related traits (low hand grip strength, appendicular lean mass, and usual walking pace) by Mendelian randomized analysis.

**Methods:**

Independent genetic variations of inflammatory cytokines and sarcopenia-related traits were selected as instrumental variables from publicly available genome-wide association studies (GWAS). The MR analysis was primarily conducted using the inverse variance-weighted (IVW) method. Sensitivity analyses included Steiger filtering and MR PRESSO, with additional assessments for heterogeneity and pleiotropy.

**Results:**

The IVW method indicated a causal relationship between Vascular Endothelial Growth Factor A (VEGF-A) and low hand grip strength (OR = 1.05654, 95% CI: 1.02453 to 1.08956, *P* = 0.00046). Additionally, Tumor Necrosis Factor-beta (TNF-β) was found to have a causal relationship with appendicular lean mass (ALM) (β = 0.04255, 95% CI: 0.02838 to 0.05672, *P* = 3.96E-09). There was no evidence suggesting a significant causal relationship between inflammatory cytokines and usual walking pace.

**Conclusion:**

Our research substantiated the causal association between inflammatory cytokines, such as VEGF-A and TNF-β, and sarcopenia. This finding may provide new avenues for future clinical treatments.

## 1 Introduction

Sarcopenia is a condition affecting the body’s skeletal muscles, marked by a gradual reduction in muscle mass and functionality that worsens as individuals age (1). In individuals aged over 60, its prevalence ranges between 10 and 27% (2). Sarcopenia is associated with a spectrum of adverse health outcomes, including fractures, falls, frailty, and mortality (3). This not only diminishes the quality of life for older individuals but also imposes a substantial clinical and economic burden on society (4).

The increasingly recognized elevation of inflammatory status with aging has been shown to be a key factor in triggering or promoting accelerated aging (5), leading to age-related conditions such as sarcopenia, atherosclerosis, type 2 diabetes, and Alzheimer’s disease (6, 7). However, inflammation is not entirely detrimental; moderate inflammatory stimulation can activate secondary adaptations against the inflammatory environment (8). Previous studies indicated that the elevation of inflammatory marker levels in the blood could cause direct or indirect damage to muscle metabolism (9). This included exacerbating the loss of muscle mass and strength, as well as a decline in physical performance (10, 11). However, previous research findings might be subject to confounding variables or reverse causation (12), and it remains uncertain whether targeting inflammation can be regarded as a novel therapeutic strategy.

Mendelian Randomization (MR) is a method utilizing genetic variations as instrumental variables (IVs) to deduce causal connections between exposures and outcomes. As alleles are randomly allocated during meiotic division, MR can mitigate biases arising from confounding variables and reverse causation, often encountered in conventional studies (13). This study employed integrated data from recently published genome-wide association studies (GWAS) on 91 inflammatory cytokines for MR analysis. The purpose was to investigate the associations between these genetic variations and sarcopenia-related traits.

## 2 Materials and methods

### 2.1 Study design

**FIGURE 1:**
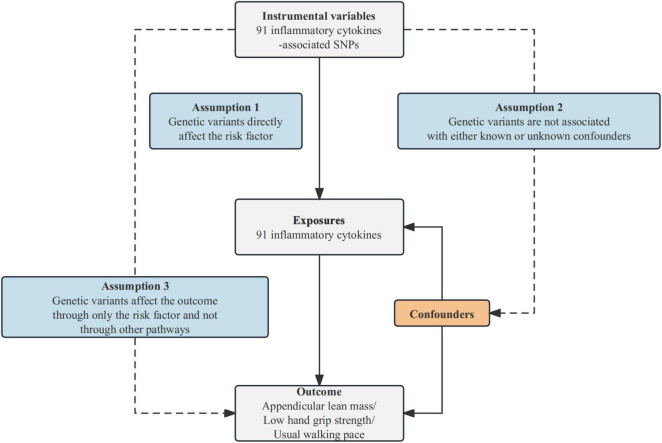
Schematic diagram of the MR analysis. SNPs stand for single-nucleotide polymorphisms. The dashed lines symbolize potential causal connections between variables, potentially indicating breaches in the assumptions of Mendelian randomization.

This study utilizes MR analysis to examine the causal relationships between inflammatory cytokines and sarcopenia-related traits, contingent upon three fundamental assumptions. First, IVs should be associated with the risk factor (91 inflammatory cytokines). Second, these genetic variants should not be associated with confounding factors. Third, IVs should influence the outcome risk (appendicular lean mass/low hand grip strength/usual walking pace) solely through the risk factor, without involving other pathways. The comprehensive design of this study can be found in Figure 1.

### 2.2 Instrumental variables selection

This study employed a series of quality control measures to identify IVs that conform to the three aforementioned assumptions (13). (1) We initially chose single nucleotide polymorphisms (SNPs) that met the genome-wide significance threshold (*P* ≤ 5 × 10^–8^) to ensure their strong correlation with the specific exposure of interest. However, due to a limited number of SNPs meeting the criteria when using inflammatory cytokines as the exposure, we raised the threshold to *P* ≤ 5 × 10^–6^. (2) A linkage disequilibrium (LD) clumping analysis was conducted to preserve independent SNPs, with an established r^2^ threshold of 0.001 and a clumping window size of 10,000 kb. (3) We further assessed the robustness of IVs using the F-statistic, and detailed information can be found in Supplementary Table 1. SNPs with an F-statistic less than 10 were considered weak IVs and were excluded due to their potential to introduce bias into the results (14). (4) When feasible, we applied corrections for allele-discordant ambiguous SNPs and linkage-ambiguous palindrome SNPs. In cases where correction was not feasible, these ambiguous and palindrome SNPs were removed from the initially selected instrumental SNPs during the harmonization process (15). Following a thorough selection process, the remaining SNPs were utilized as instrumental variables in subsequent MR analysis.

### 2.3 Data sources

The data on circulating inflammatory cytokines were obtained from a recent genome-wide protein quantitative trait locus (pQTL) study that involved 14,824 participants and assessed 91 plasma proteins (16). The study utilized a panel capable of detecting 92 inflammation-related proteins. However, due to detection issues, Brain-Derived Neurotrophic Factor (BDNF) was removed, resulting in a total of 91 proteins included in the analysis. All data used in this study were sourced from publicly available GWAS datasets, and all participants were of European ancestry.

In 2018, the European Working Group on Sarcopenia in Older People 2 (EWGSOP2) updated the definition of sarcopenia and introduced assessment tools for its application in clinical practice and research (17). These tools involve evaluating (1) Low muscle strength, typically through Grip strength. (2) Low muscle quantity or quality, often using Appendicular lean mass (ALM) as an indicator. (3) Low physical performance, commonly measured with Gait speed. Therefore, this study employed the assessment of sarcopenia through low hand grip strength, ALM, and usual walking pace (18, 19). The GWAS data for low hand grip strength were sourced from a meta-analysis study comprising 256,523 samples and 9,336,415 SNPs (20). This study comprised 22 independent cohorts, documenting the maximum handgrip strength of 256,523 participants aged 60 years or older and defined muscle weakness (dynapenia) according to the EWGSOP definition (grip strength < 30 kg for men; < 20 kg for women). The data for ALM were extracted from the GWAS study conducted by (21), which included 450,243 samples and 18,071,518 SNPs. ALM was assessed through bioelectrical impedance analysis (BIA) to measure appendicular non-fat mass. The hereditary determination of usual walking pace data was derived from the UK Biobank dataset, which included 459,915 participants. A walking speed of ≤ 0.8 m/s was recommended by EWGSOP2 as an indicator of severe sarcopenia (17).

### 2.4 Statistical analysis

In this study, we primarily assessed the potential causal relationships between 91 circulating inflammatory cytokines and sarcopenia-related traits using the most effective and statistically powerful inverse variance-weighted method (IVW) (12). At the outset, we performed two-sample MR analyses employing all 91 inflammatory cytokines as exposures, with low hand grip strength, ALM, and usual walking pace as outcomes. Exposures with a p-value below 0.05 using the IVW method were retained for subsequent analysis. Furthermore, we employed the Weighted Median method, known for its robustness to potentially invalid instrumental variables (22), as well as the MR Egger method, which is capable of detecting and correcting for bias arising from directional pleiotropy under the InSIDE (Instrument Strength Independent of Direct Effect) assumption (23).

We conducted sensitivity analyses to ensure the robustness and validity of the results. Heterogeneity was assessed using both Cochrane’s *Q* test and the MR-PRESSO global test (24). If heterogeneity is detected (*P* < 0.05), the multiplicative random-effects IVW (IVW-MRE) method will be employed to assess the causal effect (25). Furthermore, horizontal pleiotropy was examined using MR Egger regression intercept (23). We employed the MR-PRESSO global test to assess overall horizontal pleiotropy, and in the presence of detected pleiotropy, we utilized the MR-PRESSO outlier test to identify and correct for outliers (26). To assess result stability, we employed a leave-one-out approach, sequentially excluding each SNP and testing the effects of the remaining SNPs using the IVW method (27).

In MR analysis, the use of SNPs with a higher correlation to the outcome than to the exposure may lead to a misleading inference of causality. Therefore, we applied Steiger filtering to assess the causal direction of each SNP in relation to the exposure and outcome. Effective SNPs should exhibit a significantly stronger impact on the exposure variable compared to the outcome. “*R*^2^” is commonly used to compare differences in correlation between two sets of data. When the *R*^2^ value for the exposure variable exceeds that of the outcome variable, the SNP’s effect direction is categorized as “TRUE,” indicating a lower likelihood of a reverse causal relationship (28).

Due to the high number of inflammatory cytokines used as exposures, we applied a Bonferroni correction, setting the statistical significance level at *P* < 0.00055 (0.05/91). When the *p*-value is initially significant but becomes non-significant after Bonferroni corrections, it is defined as suggestive association (29). All analyses were conducted using the TwoSample MR package in R (version 4.3.1).

## 3 Results

### 3.1 Causal effects of inflammatory cytokines on low hand grip strength

**FIGURE 2:**
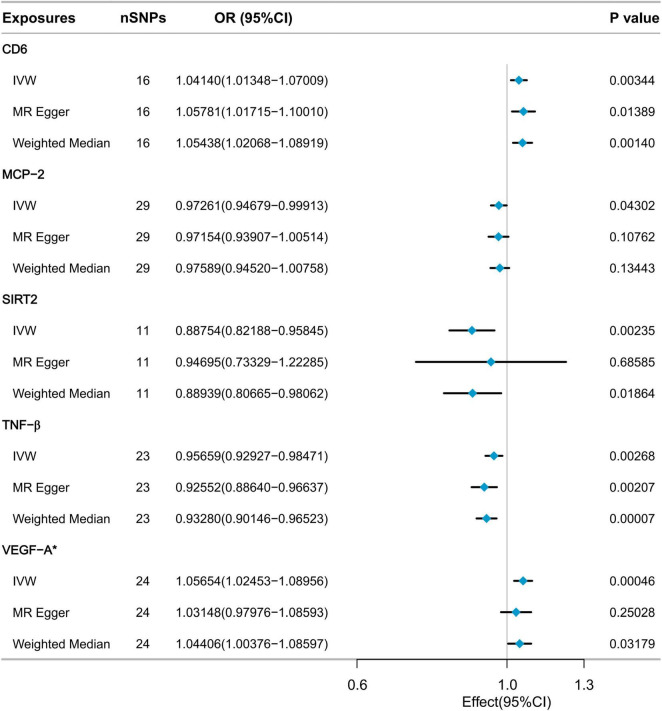
MR analysis of the effect of inflammatory cytokines on low hand grip strength.

As depicted in Figure 2, five inflammatory factors, including Cluster of Differentiation 6 (CD6), Monocyte Chemoattractant Protein-2 (MCP-2), SIR2-like protein 2 (SIRT2), and Tumor Necrosis Factor-beta (TNF-β), exhibited suggestive associations with low hand grip strength. Following Bonferroni correction, a statistically significant association was observed for Vascular Endothelial Growth Factor A (VEGF-A). The IVW method’s results indicated that a genetically determined higher level of VEGF-A (increasing by one SD) was associated with a 5.654% increased risk of low hand grip strength (OR = 1.05654, 95% CI: 1.02453 to 1.08956, *P* = 0.00046). The MR Egger method and the Weighted Median method did not reveal significant statistical associations, but they indicated a consistent trend of findings. All SNP F-statistics were greater than 10, indicating that weak IVs is unlikely to be significant (Supplementary Table 1).

Sensitivity analysis confirmed the absence of heterogeneity in the VEGF-A results (Cochrane’s *Q* test *P* = 0.530). Furthermore, there was no evidence to suggest pleiotropic effects (MR-Egger intercept test *P* = 0.266, MR-PRESSO global test *P* = 0.571). The MR-PRESSO outlier test did not detect any outliers (Supplementary Table 2). Leave-one-out analysis results indicated that no single SNP had a significant impact on the causal relationship between VEGF-A and low hand grip strength (Supplementary Figure 1). The Steiger filtering results showed that the direction of all SNPs was “TRUE” (Supplementary Table 3).

### 3.2 Causal effects of inflammatory cytokines on appendicular lean mass

**FIGURE 3:**
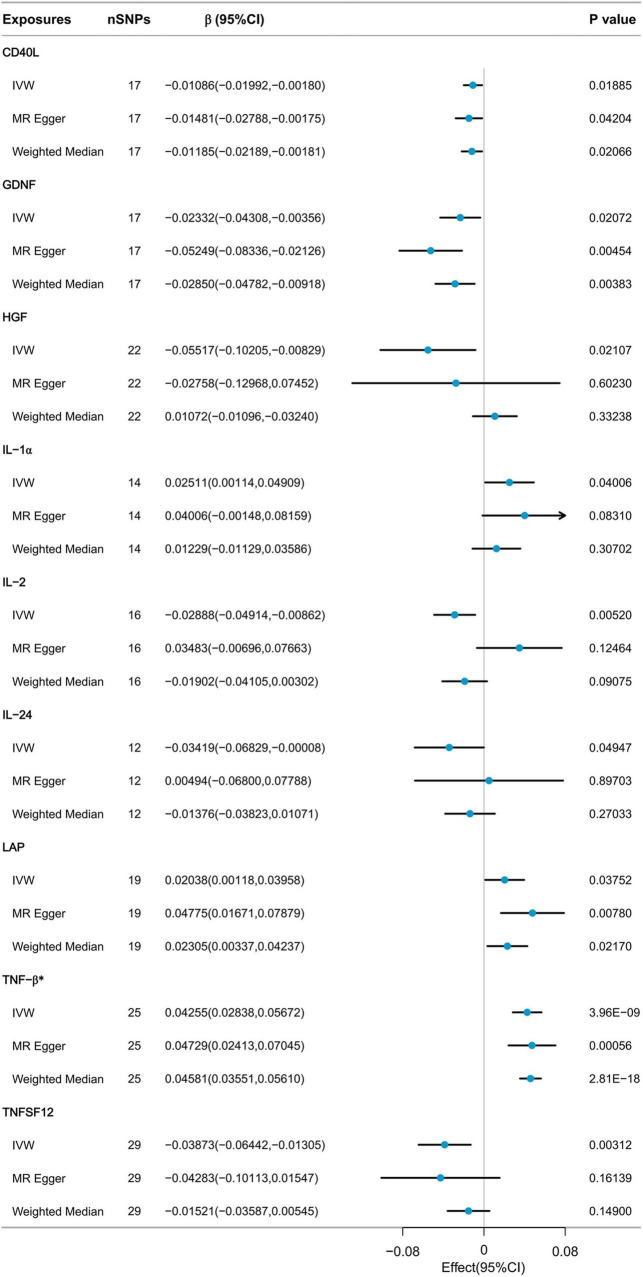
MR analysis of the effect of inflammatory cytokines on ALM.

Nine cytokines, including Cluster of Differentiation 40 Ligand (CD40L), Glial Cell Line-Derived Neurotrophic Factor (GDNF), Hepatocyte Growth Factor (HGF), Interleukin-1 alpha (IL-1α), Interleukin-2 (IL-2), Interleukin-24 (IL-24), Latency-Associated Peptide (LAP), Tumor Necrosis Factor-beta (TNF-β), and Tumor Necrosis Factor Ligand Superfamily Member 12 (TNFSF12), were selected with p-values below 0.05 using the IVW method from the initial pool of 91 inflammatory cytokines (Figure 3). According to the results of the Bonferroni correction, the IVW method (β = 0.04255, 95% CI: 0.02838 to 0.05672, *P* = 3.96E-09) indicates a statistically significant causal relationship between genetically predicted TNF-β and ALM. The Weighted Median method (β = 0.04581, 95% CI: 0.03551 to 0.05610, *P* = 2.81E-18) also demonstrated a significant statistical association. MR Egger analysis revealed a similar trend of findings. Following the calculations, it was confirmed that the F-statistic for each SNP exceeded 10 (Supplementary Table 1). As a result, the likelihood of weak IVs significantly affecting the results is relatively low.

Sensitivity analysis revealed heterogeneity in the results regarding the relationship between TNF-β and ALM (Cochrane’s *Q* test *P* < 0.001). The MR-Egger intercept test did not detect horizontal pleiotropy. MR-PRESSO detected three potential outliers, leading to indications of potential pleiotropy in the MR-PRESSO global test. However, after correcting for these outliers, the results remained similar. Further details of the results can be found in Supplementary Table 2. Due to the presence of heterogeneity, we proceeded to further assess the causal relationship between TNF-β and ALM using the IVW-MRE method (β = 0.04255, 95% CI: 0.02889 to 0.05621, *P* = 3.96E-09), and the results still indicated a significant statistical association. The results of the leave-one-out analysis indicated that no individual SNP could exert a complete influence on the causal relationship between TNF-β and ALM (Supplementary Figure 2). The results of Steiger filtering indicated that the direction of all SNPs was “TRUE” (Supplementary Table 4).

### 3.3 Causal effects of inflammatory cytokines on usual walking pace

**FIGURE 4:**
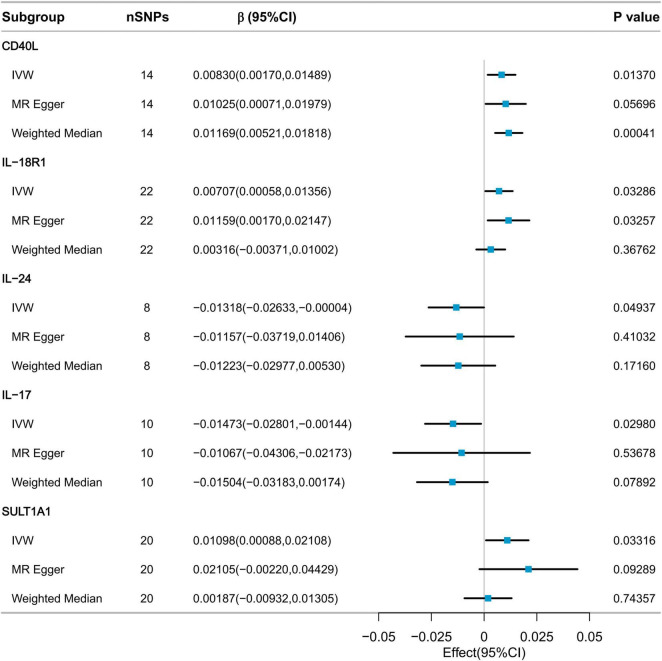
MR analysis of the effect of inflammatory cytokines on usual walking pace.

The IVW method was employed to collectively identify five inflammatory factors with p-values less than 0.05. These factors included CD40L, Interleukin-18 Receptor 1 (IL-18R1), Interleukin-24 (IL-24), Interleukin-7 (IL-7), and Sulfotransferase 1A1 (SULT1A1). However, after Bonferroni correction, none of the inflammatory factors have been demonstrated to have a significant causal relationship with usual walking pace (Figure 4). CD40L (β = 0.00830, 95% CI: 0.00170 to 0.01489, *P* = 0.0137), IL-18R1 (β = 0.00707, 95% CI: 0.00058 to 0.01356, *P* = 0.03286), IL-24 (β = −0.01318, 95% CI: −0.01318 to −0.00004, *P* = 0.04937), IL-7 (β = −0.01473, 95% CI: −0.02801 to −0.00144, *P* = 0.0298), and SULT1A1 (β = 0.01098, 95% CI: 0.00088 to 0.02108, *P* = 0.03316).

## 4 Discussion

This study applied Mendelian randomization analysis to examine the causal connection between 91 circulating inflammatory cytokines and sarcopenia-related traits. We identified a positive causal relationship between VEGF-A and low handgrip strength, as well as a genetic predisposition to elevated levels of TNF-β was associated with an increase in appendicular lean mass. No evidence of a reverse causal relationship was found. However, no significant causal relationship was observed between the inflammatory cytokines and usual walking pace. Several inflammatory cytokines, such as CD6, HGF, and IL-7, have demonstrated suggestive associations with sarcopenia-related traits. To the best of our knowledge, this is the first study to employ GWAS statistical data in assessing the causal relationships between inflammatory cytokines and sarcopenia-related traits.

An increasing body of evidence suggested that inflammatory cytokines had a detrimental impact on muscle metabolism (9), emphasizing the role of chronic inflammation clearance in the pathogenesis of sarcopenia (30). Observational studies have suggested that an increase in the levels of inflammatory biomarkers in the bloodstream is associated with a loss of muscle mass and strength, as well as a decline in physical activity. VEGF-A serves as a critical regulator of vascular development (31), but its influence on sarcopenia remains uncertain. Previous research has indicated that aging in multiple organs, including skeletal muscle, is associated with inadequate VEGF signaling (32). Adult mice lacking VEGF in skeletal muscle display significant intolerance to aerobic exercise, while VEGF-A can promote muscle regeneration in aging mice (33, 34). VEGF-A may play a protective role in skeletal muscle. Interestingly, our study uncovered unexpected findings, demonstrating a positive causal relationship between VEGF-A and low handgrip strength. Specifically, an increase in VEGF-A levels is linked to a higher risk of low handgrip strength in older individuals. A study conducted on VEGF-A in the hind limb skeletal muscles of both normal and ischemic rabbits discovered that prolonged VEGF-A expression can result in abnormal muscle angiogenesis and fibrosis (35). This supports the findings of our study. Research suggests that the optimal VEGF dosage, despite its potent angiogenic potential, lies within a narrow range, as excessive production results in the formation of disordered, irregularly sized vessels with reduced wall permeability (36, 37). Some researchers recommend careful regulation of VEGF expression because VEGF-induced angiogenesis may be dose-dependent. Prolonged irregular expression has been associated with a high incidence of developmental abnormalities or mortality in mice, making overexpression detrimental (38, 39). It’s worth noting that while VEGF can induce mature blood vessels at the appropriate dosage, the balance between clinical benefits and toxicity may vary due to genetic susceptibility, age, and disease status (40). This influence might also be applicable to skeletal muscle. All the participants in our study with low hand grip strength were individuals aged over 60, and age differences might influence the results. Therefore, it is imperative to further investigate the potential adverse effects of VEGF-A expression on muscle metabolism in future clinical practice and research, while also taking into account the potential differential regulation of VEGF-A expression in aging.

Tumor necrosis factor-beta (TNF-β), composed of 205 amino acids, induces the generation and release of inflammatory factors. It targets TNFR2, activating signal transduction pathways crucial for normal immune function, also known as lymphotoxin (LT)-α (41). Our research suggested that upregulation of TNF-β contributes to the enhancement of muscle mass. Previous studies on the relationship between TNF-β and muscle metabolism are limited. However, it forms a heterotrimer with LTβ and binds to the LTβ receptor. In a study focused on idiopathic inflammatory myopathies, a robust upregulation of LTβ was observed in regenerating muscle fibers (42). Previous research has primarily focused on the correlation between inflammatory cytokines and sarcopenia, with the majority adopting cross-sectional study designs. This indicates that these studies cannot definitively establish a causal relationship between inflammatory cytokines and sarcopenia (4). Our study indicated a causal relationship between VEGF-A and low handgrip strength, as well as between TNF-β and ALM. Simultaneously, results contrary to previous research findings were also uncovered. For instance, previous studies had suggested that elevated IL-6 levels in the older people were associated with an increased risk of muscle strength loss (43). Prolonged exposure to high IL-6 levels was linked to muscle atrophy and could directly regulate muscle catabolism, resulting in diminished muscle strength (44). Research has identified a significant correlation between elevated levels of IL-8 in serum and the development of cancer cachexia and muscle wasting in pancreatic cancer patients (45). Similar results were observed in a study involving older individuals in the United Kingdom (46). However, our MR analysis did not identify a causal relationship between IL-6 or IL-8 and sarcopenia-related traits. Due to the immense complexity of the inflammatory response and the multifactorial nature of sarcopenia, studying the association between a single inflammatory marker and sarcopenia is insufficient. In the future, comprehensive research on multiple inflammation markers will be necessary to enhance the sensitivity and specificity of diagnosis (8).

This study has several strengths. We utilized the latest large-scale data encompassing 91 inflammatory inflammatory cytokines for MR analysis. This effectively mitigated the influences of confounding factors and reverse causation, and a series of sensitivity analyses were conducted to ensure the reliability of the results. However, our study has the following limitations. Firstly, to mitigate bias, all data in this study originated from populations of European ancestry, limiting the generalizability of the results to other populations. Secondly, MR analysis typically examines the lifelong impact of risk factors on outcomes, thereby lacking the ability to identify effects at different stages. Lastly, we were unable to rule out non-linear causal relationships in the results.

## 5 Conclusion

This study demonstrated a causal relationship between the circulating inflammatory cytokine VEGF-A and an elevated risk of low hand grip strength in older adults. Additionally, it suggested that TNF-β may contribute to an increase in appendicular lean mass. No causal relationship was identified between inflammatory cytokines and usual walking pace. This may offer new insights into treating sarcopenia, but further research is needed to validate these findings in the future.

## Data Availability

The original contributions presented in the study are included in the article/Supplementary material, further inquiries can be directed to the corresponding author.

## Supplementary material

Supplementary Figure 1 MR leave-one-out senstivity analysis for VEGF-A on low hand grip strength.

Supplementary Figure 2 MR leave-one-out senstivity analysis for TNF-β on appendicular lean mass.

## References

[1] Yuan S Larsson S. Epidemiology of sarcopenia: Prevalence, risk factors, and consequences. *Metabolism.* (2023) 144:155533. 10.1016/j.metabol.2023.155533 36907247

[2] Petermann-Rocha F Balntzi V Gray S Lara J Ho F Pell J Global prevalence of sarcopenia and severe sarcopenia: A systematic review and meta-analysis. *J Cachexia Sarcopenia Muscle.* (2022) 13:86–99. 10.1002/jcsm.12783 34816624 PMC8818604

[3] Cruz-Jentoft A Sayer A. Sarcopenia. *Lancet.* (2019) 393:2636–46. 10.1016/S0140-6736(19)31138-9 31171417

[4] Pan L Xie W Fu X Lu W Jin H Lai J Inflammation and sarcopenia: A focus on circulating inflammatory cytokines. *Exp Gerontol.* (2021) 154:111544. 10.1016/j.exger.2021.111544 34478826

[5] Pawelec G Goldeck D Derhovanessian E. Inflammation, ageing and chronic disease. *Curr Opin Immunol.* (2014) 29:23–8. 10.1016/j.coi.2014.03.007 24762450

[6] Duchesne E Dufresne S Dumont N. Impact of inflammation and anti-inflammatory modalities on skeletal muscle healing: From fundamental research to the clinic. *Phys Ther.* (2017) 97:807–17. 10.1093/ptj/pzx056 28789470

[7] JafariNasabian P Inglis J Reilly W Kelly O Ilich J. Aging human body: Changes in bone, muscle and body fat with consequent changes in nutrient intake. *J Endocrinol.* (2017) 234:R37–51. 10.1530/JOE-16-0603 28442508

[8] Furman D Campisi J Verdin E Carrera-Bastos P Targ S Franceschi C Chronic inflammation in the etiology of disease across the life span. *Nat Med.* (2019) 25:1822–32. 10.1038/s41591-019-0675-0 31806905 PMC7147972

[9] Chung H Cesari M Anton S Marzetti E Giovannini S Seo A Molecular inflammation: Underpinnings of aging and age-related diseases. *Ageing Res Rev.* (2009) 8:18–30. 10.1016/j.arr.2008.07.002 18692159 PMC3782993

[10] Visser M Pahor M Taaffe D Goodpaster B Simonsick E Newman A Relationship of interleukin-6 and tumor necrosis factor-alpha with muscle mass and muscle strength in elderly men and women: The health ABC study. *J Gerontol A Biol Sci Med Sci.* (2002) 57:M326–32. 10.1093/gerona/57.5.m326 11983728

[11] Cesari M Penninx B Pahor M Lauretani F Corsi A Rhys Williams G Inflammatory markers and physical performance in older persons: The InCHIANTI study. *J Gerontol A Biol Sci Med Sci.* (2004) 59:242–8. 10.1093/gerona/59.3.m242 15031308

[12] Burgess S Butterworth A Thompson S. Mendelian randomization analysis with multiple genetic variants using summarized data. *Genet Epidemiol.* (2013) 37:658–65. 10.1002/gepi.21758 24114802 PMC4377079

[13] Burgess S Scott R Timpson N Davey Smith G Thompson S Consortium E. Using published data in Mendelian randomization: A blueprint for efficient identification of causal risk factors. *Eur J Epidemiol.* (2015) 30:543–52. 10.1007/s10654-015-0011-z 25773750 PMC4516908

[14] Papadimitriou N Dimou N Tsilidis K Banbury B Martin R Lewis S Physical activity and risks of breast and colorectal cancer: A Mendelian randomisation analysis. *Nat Commun.* (2020) 11:597. 10.1038/s41467-020-14389-8 32001714 PMC6992637

[15] Wu F Huang Y Hu J Shao Z. Mendelian randomization study of inflammatory bowel disease and bone mineral density. *BMC Med.* (2020) 18:312. 10.1186/s12916-020-01778-5 33167994 PMC7654011

[16] Zhao J Stacey D Eriksson N Macdonald-Dunlop E Hedman A Kalnapenkis A Genetics of circulating inflammatory proteins identifies drivers of immune-mediated disease risk and therapeutic targets. *Nat Immunol.* (2023) 24:1540–51. 10.1038/s41590-023-01588-w 37563310 PMC10457199

[17] Cruz-Jentoft A Bahat G Bauer J Boirie Y Bruyere O Cederholm T Sarcopenia: Revised European consensus on definition and diagnosis. *Age Ageing.* (2019) 48:16–31. 10.1093/ageing/afy169 30312372 PMC6322506

[18] Ye C Kong L Wang Y Zheng J Xu M Xu Y Causal associations of sarcopenia-related traits with cardiometabolic disease and Alzheimer’s disease and the mediating role of insulin resistance: A Mendelian randomization study. *Aging Cell.* (2023) 22:e13923. 10.1111/acel.13923 37403750 PMC10497819

[19] Xia X Xiang S Hua L Sun Q Wang R. The relationship between lifestyles and sarcopenia-related traits: A two-sample Mendelian randomization study. *Arch Gerontol Geriatr.* (2023) 116:105169. 10.1016/j.archger.2023.105169 37657206

[20] Jones G Trajanoska K Santanasto A Stringa N Kuo C Atkins J Genome-wide meta-analysis of muscle weakness identifies 15 susceptibility loci in older men and women. *Nat Commun.* (2021) 12:654. 10.1038/s41467-021-20918-w 33510174 PMC7844411

[21] Pei Y Liu Y Yang X Zhang H Feng G Wei X The genetic architecture of appendicular lean mass characterized by association analysis in the UK Biobank study. *Commun Biol.* (2020) 3:608. 10.1038/s42003-020-01334-0 33097823 PMC7585446

[22] Bowden J Davey Smith G Haycock P Burgess S. Consistent estimation in Mendelian randomization with some invalid instruments using a weighted median estimator. *Genet Epidemiol.* (2016) 40:304–14. 10.1002/gepi.21965 27061298 PMC4849733

[23] Bowden J Davey Smith G Burgess S. Mendelian randomization with invalid instruments: Effect estimation and bias detection through Egger regression. *Int J Epidemiol.* (2015) 44:512–25. 10.1093/ije/dyv080 26050253 PMC4469799

[24] Emdin C Khera A Kathiresan S. Mendelian Randomization. *JAMA.* (2017) 318:1925–6. 10.1001/jama.2017.17219 29164242

[25] Chen Z Chen Z Jin X. Mendelian randomization supports causality between overweight status and accelerated aging. *Aging Cell.* (2023) 22:e13899. 10.1111/acel.13899 37277933 PMC10410004

[26] Verbanck M Chen C Neale B Do R. Detection of widespread horizontal pleiotropy in causal relationships inferred from Mendelian randomization between complex traits and diseases. *Nat Genet.* (2018) 50:693–8. 10.1038/s41588-018-0099-7 29686387 PMC6083837

[27] Xiang M Wang Y Gao Z Wang J Chen Q Sun Z Exploring causal correlations between inflammatory cytokines and systemic lupus erythematosus: A Mendelian randomization. *Front Immunol.* (2022) 13:985729. 10.3389/fimmu.2022.985729 36741410 PMC9893779

[28] Hemani G Tilling K Davey Smith G. Orienting the causal relationship between imprecisely measured traits using GWAS summary data. *PLoS Genet.* (2017) 13:e1007081. 10.1371/journal.pgen.1007081 29149188 PMC5711033

[29] Georgakis M Gill D Rannikmae K Traylor M Anderson C Lee J Genetically determined levels of circulating cytokines and risk of stroke. *Circulation.* (2019) 139:256–68. 10.1161/CIRCULATIONAHA.118.035905 30586705 PMC7477819

[30] Liang Z Zhang T Liu H Li Z Peng L Wang C Inflammaging: The ground for sarcopenia? *Exp Gerontol.* (2022) 168:111931. 10.1016/j.exger.2022.111931 35985553

[31] Holmes D Zachary I. The vascular endothelial growth factor (VEGF) family: Angiogenic factors in health and disease. *Genome Biol.* (2005) 6:209. 10.1186/gb-2005-6-2-209 15693956 PMC551528

[32] Grunewald M Kumar S Sharife H Volinsky E Gileles-Hillel A Licht T Counteracting age-related VEGF signaling insufficiency promotes healthy aging and extends life span. *Science.* (2021) 373:eabc8479. 10.1126/science.abc8479 34326210

[33] Olfert I Howlett R Tang K Dalton N Gu Y Peterson K Muscle-specific VEGF deficiency greatly reduces exercise endurance in mice. *J Physiol.* (2009) 587:1755–67. 10.1113/jphysiol.2008.164384 19237429 PMC2683962

[34] Endo Y Hwang C Zhang Y Olumi S Koh D Zhu C VEGFA promotes skeletal muscle regeneration in aging. *Adv Biol (Weinh).* (2023) 7:e2200320. 10.1002/adbi.202200320 36988414 PMC10539483

[35] Karvinen H Pasanen E Rissanen T Korpisalo P Vahakangas E Jazwa A Long-term VEGF-A expression promotes aberrant angiogenesis and fibrosis in skeletal muscle. *Gene Ther.* (2011) 18:1166–72. 10.1038/gt.2011.66 21562595

[36] Ozawa C Banfi A Glazer N Thurston G Springer M Kraft P Microenvironmental VEGF concentration, not total dose, determines a threshold between normal and aberrant angiogenesis. *J Clin Invest.* (2004) 113:516–27. 10.1172/JCI18420 14966561 PMC338257

[37] Kennedy D Wheatley A McCullagh KJA. VEGF-A and FGF4 engineered C2C12 myoblasts and angiogenesis in the chick chorioallantoic membrane. *Biomedicines.* (2022) 10:1781. 10.3390/biomedicines10081781 35892681 PMC9330725

[38] Springer M Chen A Kraft P Bednarski M Blau HM. VEGF gene delivery to muscle: Potential role for vasculogenesis in adults. *Mol Cell.* (1998) 2:549–58. 10.1016/s1097-2765(00)80154-9 9844628

[39] Lee R Springer M Blanco-Bose W Shaw R Ursell P Blau HM. VEGF gene delivery to myocardium: Deleterious effects of unregulated expression. *Circulation.* (2000) 102:898–901. 10.1161/01.cir.102.8.898 10952959

[40] Blau H Banfi A. The well-tempered vessel. *Nat Med.* (2001) 7:532–4. 10.1038/87850 11329048

[41] Buhrmann C Shayan P Aggarwal B Shakibaei M. Evidence that TNF-beta (lymphotoxin alpha) can activate the inflammatory environment in human chondrocytes. *Arthritis Res Ther.* (2013) 15:R202. 10.1186/ar4393 24283517 PMC3979010

[42] Creus K De Paepe B Weis J De Bleecker J. The multifaceted character of lymphotoxin beta in inflammatory myopathies and muscular dystrophies. *Neuromuscul Disord.* (2012) 22:712–9. 10.1016/j.nmd.2012.04.012 22652080

[43] Schaap L Pluijm S Deeg D Visser M. Inflammatory markers and loss of muscle mass (sarcopenia) and strength. *Am J Med.* (2006) 119:526.e9–17. 10.1016/j.amjmed.2005.10.049 16750969

[44] Belizario J Fontes-Oliveira C Borges J Kashiabara J Vannier E. Skeletal muscle wasting and renewal: A pivotal role of myokine IL-6. *Springerplus.* (2016) 5:619. 10.1186/s40064-016-2197-2 27330885 PMC4870483

[45] Hou Y Wang C Chao Y Chen H Wang H Tung H Elevated serum interleukin-8 level correlates with cancer-related cachexia and sarcopenia: An indicator for pancreatic cancer outcomes. *J Clin Med.* (2018) 7:502. 10.3390/jcm7120502 30513776 PMC6306800

[46] Westbury L Fuggle N Syddall H Duggal N Shaw S Maslin K Relationships between markers of inflammation and muscle mass, strength and function: Findings from the hertfordshire cohort study. *Calcif Tissue Int.* (2018) 102:287–95. 10.1007/s00223-017-0354-4 29101476 PMC5818589

