# Supplementary material for: Circulating inflammatory cytokines and sarcopenia-related traits: a mendelian randomization analysis
Source: Front Med (Lausanne). 2024 Aug 13;11:1351376. doi: 10.3389/fmed.2024.1351376 (PMC11347448; doi:10.3389/fmed.2024.1351376)
Supplement: Supplementary file 1 [file Table_1.DOC]

**Table S1.** The F statistics for each instrument-exposure association in MR study.

The F statistic was defined by the formula: F = R2/(1-R2) × (N-k-1)/k. Here, R2 represented the proportion of exposure variance explained by the IVs, N denoted the sample size of exposure, and k represented the count of IVs.

| Phenotype | SNP | EAF | beta | SE (beta) | N | R^2^ | F statistic |
| --- | --- | --- | --- | --- | --- | --- | --- |
| VEGF−A | rs2050256 | 0.8126 | -0.0734 | 0.0152 | 14739 | 1.58E-03 | 23.31556847 |
| VEGF−A | rs61818787 | 0.8114 | -0.0759 | 0.0148 | 14732 | 1.78E-03 | 26.2966943 |
| VEGF−A | rs183596950 | 0.6438 | -0.07 | 0.0152 | 11538 | 1.83E-03 | 21.20477248 |
| VEGF−A | rs7688836 | 0.2065 | -0.0685 | 0.0145 | 14742 | 1.51E-03 | 22.31445145 |
| VEGF−A | rs16883415 | 0.9797 | -0.22 | 0.0457 | 14744 | 1.57E-03 | 23.17149453 |
| VEGF−A | rs114694170 | 0.9462 | -0.1625 | 0.0267 | 14728 | 2.51E-03 | 37.03609834 |
| VEGF−A | rs11953790 | 0.028 | -0.1842 | 0.0374 | 14730 | 1.64E-03 | 24.25364832 |
| VEGF−A | rs9369434 | 0.4233 | -0.3686 | 0.012 | 14295 | 6.19E-02 | 943.381605 |
| VEGF−A | rs9349270 | 0.1166 | 0.1055 | 0.0188 | 14296 | 2.20E-03 | 31.48679517 |
| VEGF−A | rs7808444 | 0.6119 | -0.0628 | 0.0137 | 14296 | 1.47E-03 | 21.00958101 |
| VEGF−A | rs1208181 | 0.8309 | -0.079 | 0.0166 | 12855 | 1.76E-03 | 22.64490135 |
| VEGF−A | rs4875579 | 0.8345 | -0.0741 | 0.0158 | 14296 | 1.54E-03 | 21.9918356 |
| VEGF−A | rs117976551 | 0.9739 | -0.2163 | 0.0449 | 13809 | 1.68E-03 | 23.20371123 |
| VEGF−A | rs6993770 | 0.7319 | 0.1231 | 0.0134 | 14296 | 5.87E-03 | 84.38120974 |
| VEGF−A | rs145703410 | 0.0153 | -0.2475 | 0.0539 | 14296 | 1.47E-03 | 21.08201483 |
| VEGF−A | rs10822155 | 0.4155 | 0.0838 | 0.0117 | 14744 | 3.47E-03 | 51.29291707 |
| VEGF−A | rs10840177 | 0.7087 | -0.0683 | 0.0142 | 13231 | 1.75E-03 | 23.13124804 |
| VEGF−A | rs34536806 | 0.9024 | 0.0992 | 0.0208 | 14296 | 1.59E-03 | 22.74238004 |
| VEGF−A | rs6582666 | 0.9341 | -0.1288 | 0.0265 | 11791 | 2.00E-03 | 23.61926107 |
| VEGF−A | rs145846107 | 0.0162 | 0.2736 | 0.0582 | 13422 | 1.64E-03 | 22.09639873 |
| VEGF−A | rs138648222 | 0.0248 | 0.1981 | 0.0397 | 14739 | 1.69E-03 | 24.89596715 |
| VEGF−A | rs144037665 | 0.9871 | -0.2779 | 0.0603 | 11969 | 1.77E-03 | 21.23586195 |
| VEGF−A | rs1203834 | 0.069 | 0.1174 | 0.0253 | 13232 | 1.62E-03 | 21.52927987 |
| VEGF−A | rs62640435 | 0.296 | -0.0671 | 0.0127 | 14292 | 1.95E-03 | 27.91109145 |
| TNF−β | rs77897196 | 0.9711 | -0.1917 | 0.041 | 11792 | 1.85E-03 | 21.85761877 |
| TNF−β | rs12743015 | 0.029 | -0.1918 | 0.0403 | 11784 | 1.92E-03 | 22.64714173 |
| TNF−β | rs7583622 | 0.2098 | 0.0748 | 0.0161 | 11769 | 1.83E-03 | 21.58130161 |
| TNF−β | rs112317147 | 0.0534 | -0.1441 | 0.0309 | 11344 | 1.91E-03 | 21.74374909 |
| TNF−β | rs16852556 | 0.0511 | 0.1514 | 0.0317 | 11344 | 2.01E-03 | 22.80639548 |
| TNF−β | rs192303297 | 0.0118 | -0.3872 | 0.0842 | 7843 | 2.69E-03 | 21.14149784 |
| TNF−β | rs113244922 | 0.0237 | 0.2324 | 0.0499 | 10470 | 2.07E-03 | 21.68643618 |
| TNF−β | rs114250919 | 0.9743 | 0.2469 | 0.049 | 11305 | 2.24E-03 | 25.38476696 |
| TNF−β | rs76913603 | 0.967 | -0.1741 | 0.0379 | 11782 | 1.79E-03 | 21.09819949 |
| TNF−β | rs72854560 | 0.0156 | -0.2968 | 0.0602 | 11344 | 2.14E-03 | 24.30290761 |
| TNF−β | rs2074475 | 0.9618 | 0.2308 | 0.0421 | 11792 | 2.54E-03 | 30.04925795 |
| TNF−β | rs2523882 | 0.2883 | -0.1991 | 0.0144 | 11792 | 1.60E-02 | 191.1366062 |
| TNF−β | rs2904602 | 0.1165 | -0.625 | 0.0196 | 11791 | 7.94E-02 | 1016.65645 |
| TNF−β | rs542608643 | 0.0294 | -0.3554 | 0.0467 | 8387 | 6.86E-03 | 57.90252593 |
| TNF−β | rs9267798 | 0.0834 | -0.1199 | 0.0238 | 11792 | 2.15E-03 | 25.3752767 |
| TNF−β | rs74363807 | 0.0218 | 0.2496 | 0.0532 | 11344 | 1.94E-03 | 22.00844317 |
| TNF−β | rs138471526 | 0.0137 | -0.3418 | 0.0735 | 9983 | 2.16E-03 | 21.62133088 |
| TNF−β | rs139511528 | 0.0294 | -0.2485 | 0.0521 | 10280 | 2.21E-03 | 22.74536122 |
| TNF−β | rs2364485 | 0.1645 | 0.1759 | 0.0189 | 11344 | 7.58E-03 | 86.60271267 |
| TNF−β | rs147423408 | 0.0415 | 0.1732 | 0.035 | 11344 | 2.15E-03 | 24.48404177 |
| TNF−β | rs537860389 | 0.9731 | -0.229 | 0.0491 | 9240 | 2.35E-03 | 21.74773173 |
| TNF−β | rs143019966 | 0.9751 | 0.2122 | 0.0451 | 10913 | 2.02E-03 | 22.13390675 |
| TNF−β | rs12883833 | 0.0934 | -0.1252 | 0.0259 | 9259 | 2.52E-03 | 23.36228455 |
| TNF−β | rs28700215 | 0.5824 | -0.0626 | 0.0136 | 11344 | 1.86E-03 | 21.1833321 |
| TNF−β | rs55914821 | 0.7583 | 0.0748 | 0.0159 | 11344 | 1.95E-03 | 22.12750116 |
