# Supplementary material for: Circulating inflammatory cytokines and sarcopenia-related traits: a mendelian randomization analysis
Source: Front Med (Lausanne). 2024 Aug 13;11:1351376. doi: 10.3389/fmed.2024.1351376 (PMC11347448; doi:10.3389/fmed.2024.1351376)
Supplement: Supplementary file 2 [file Table_2.DOC]

**Table S2.** Sensitivity analysis of inflammatory cytokines on appendicular lean mass and low hand grip strength.

|  | Pleiotropy test | | | |  | Heterogeneity test | |
| --- | --- | --- | --- | --- | --- | --- | --- |
|  | MR-Egger intercept test | | MR-PRESSO global test | |  | Cochrane's Q test | |
| TNF−β on | Intercept | P | RSSobs | P |  | Q | P |
| appendicular lean mass | -0.001 | 0.613 | 127.235 | <0.001 |  | 107.408 | <0.001 |
| VEGF-A on |  |  |  |  |  |  |  |
| low hand grip strength | 0.004 | 0.266 | 24.472 | 0.571 |  | 21.841 | 0.530 |
