# Supplementary material for: Circulating inflammatory cytokines and sarcopenia-related traits: a mendelian randomization analysis
Source: Front Med (Lausanne). 2024 Aug 13;11:1351376. doi: 10.3389/fmed.2024.1351376 (PMC11347448; doi:10.3389/fmed.2024.1351376)
Supplement: Supplementary file 3 [file Table_3.DOC]

**Table S3. Steiger filtering analysis of VEGF-A and low hand grip strength.**

| SNP | rsq.exposure | rsq.outcome | steiger_dir | steiger_pval |
| --- | --- | --- | --- | --- |
| rs10822155 | 0.003467778 | 1.34E-05 | TRUE | 6.61E-11 |
| rs10840177 | 0.001745737 | 1.71E-05 | TRUE | 2.39E-05 |
| rs114694170 | 0.002509045 | 6.84E-07 | TRUE | 5.94E-09 |
| rs117976551 | 0.001677999 | 4.08E-06 | TRUE | 8.19E-06 |
| rs11953790 | 0.001644287 | 2.62E-06 | TRUE | 4.28E-06 |
| rs1203834 | 0.001624909 | 7.52E-07 | TRUE | 9.58E-06 |
| rs1208181 | 0.001759012 | 1.06E-07 | TRUE | 4.09E-06 |
| rs138648222 | 0.00168673 | 1.14E-05 | TRUE | 8.50E-06 |
| rs144037665 | 0.001771687 | 3.28E-06 | TRUE | 1.64E-05 |
| rs145703410 | 0.001472919 | 4.71E-08 | TRUE | 8.89E-06 |
| rs145846107 | 0.001644065 | 5.55E-07 | TRUE | 6.89E-06 |
| rs16883415 | 0.001569547 | 8.17E-06 | TRUE | 1.41E-05 |
| rs183596950 | 0.001835084 | 2.92E-07 | TRUE | 8.72E-06 |
| rs2050256 | 0.001579826 | 4.06E-10 | TRUE | 2.70E-06 |
| rs34536806 | 0.001588738 | 6.62E-06 | TRUE | 1.42E-05 |
| rs4875579 | 0.001536387 | 2.99E-06 | TRUE | 1.29E-05 |
| rs61818787 | 0.001782308 | 1.05E-05 | TRUE | 4.15E-06 |
| rs62640435 | 0.001949655 | 4.00E-06 | TRUE | 9.21E-07 |
| rs6582666 | 0.001999833 | 1.19E-05 | TRUE | 1.16E-05 |
| rs6993770 | 0.005869433 | 9.04E-07 | TRUE | 1.13E-18 |
| rs7688836 | 0.001511787 | 4.41E-06 | TRUE | 1.39E-05 |
| rs7808444 | 0.001467866 | 1.07E-06 | TRUE | 1.43E-05 |
| rs9349270 | 0.002198263 | 1.23E-05 | TRUE | 4.40E-07 |
| rs9369434 | 0.061924508 | 1.58E-05 | TRUE | 2.52E-186 |
