# Supplementary material for: Circulating inflammatory cytokines and sarcopenia-related traits: a mendelian randomization analysis
Source: Front Med (Lausanne). 2024 Aug 13;11:1351376. doi: 10.3389/fmed.2024.1351376 (PMC11347448; doi:10.3389/fmed.2024.1351376)
Supplement: Supplementary file 4 [file Table_4.DOC]

**Table S4. Steiger filtering analysis of TNF-β and appendicular lean.**

| SNP | rsq.exposure | rsq.outcome | steiger_dir | steiger_pval |
| --- | --- | --- | --- | --- |
| rs112317147 | 0.021326438 | 1.11E-05 | TRUE | 5.79E-06 |
| rs113244922 | 0.021271727 | 8.88E-08 | TRUE | 3.77E-06 |
| rs114250919 | 0.024808995 | 7.22E-06 | TRUE | 8.44E-07 |
| rs12743015 | 0.022192685 | 6.32E-07 | TRUE | 2.49E-06 |
| rs12883833 | 0.02287848 | 3.05E-06 | TRUE | 2.01E-06 |
| rs138471526 | 0.021209415 | 1.40E-06 | TRUE | 4.45E-06 |
| rs139511528 | 0.02228733 | 1.96E-07 | TRUE | 2.24E-06 |
| rs143019966 | 0.021700951 | 5.72E-07 | TRUE | 3.22E-06 |
| rs147423408 | 0.023949768 | 3.79E-07 | TRUE | 9.52E-07 |
| rs16852556 | 0.0223454 | 3.38E-07 | TRUE | 2.22E-06 |
| rs192303297 | 0.0207496 | 7.72E-07 | TRUE | 5.43E-06 |
| rs2074475 | 0.029234208 | 6.67E-07 | TRUE | 5.94E-08 |
| rs2364485 | 0.079860361 | 3.29E-09 | TRUE | 5.16E-20 |
| rs2523882 | 0.1607585 | 0.000234328 | TRUE | 3.71E-38 |
| rs28700215 | 0.020788203 | 2.29E-05 | TRUE | 9.49E-06 |
| rs2904602 | 0.504672587 | 0.000243257 | TRUE | 1.14E-166 |
| rs537860389 | 0.021331099 | 4.97E-07 | TRUE | 3.88E-06 |
| rs542608643 | 0.054849362 | 2.16E-05 | TRUE | 1.59E-13 |
| rs55914821 | 0.021694659 | 3.06E-06 | TRUE | 3.76E-06 |
| rs72854560 | 0.023776799 | 2.07E-05 | TRUE | 1.94E-06 |
| rs74363807 | 0.021580449 | 1.60E-07 | TRUE | 3.25E-06 |
| rs7583622 | 0.021170349 | 1.68E-08 | TRUE | 3.88E-06 |
| rs76913603 | 0.020706255 | 1.84E-06 | TRUE | 5.97E-06 |
| rs77897196 | 0.021435587 | 7.92E-06 | TRUE | 5.06E-06 |
| rs9267798 | 0.024799773 | 8.88E-08 | TRUE | 5.76E-07 |
