# Supplementary figures and images for: Circulating inflammatory cytokines and sarcopenia-related traits: a mendelian randomization analysis
Source: Front Med (Lausanne). 2024 Aug 13;11:1351376. doi: 10.3389/fmed.2024.1351376 (PMC11347448; doi:10.3389/fmed.2024.1351376)

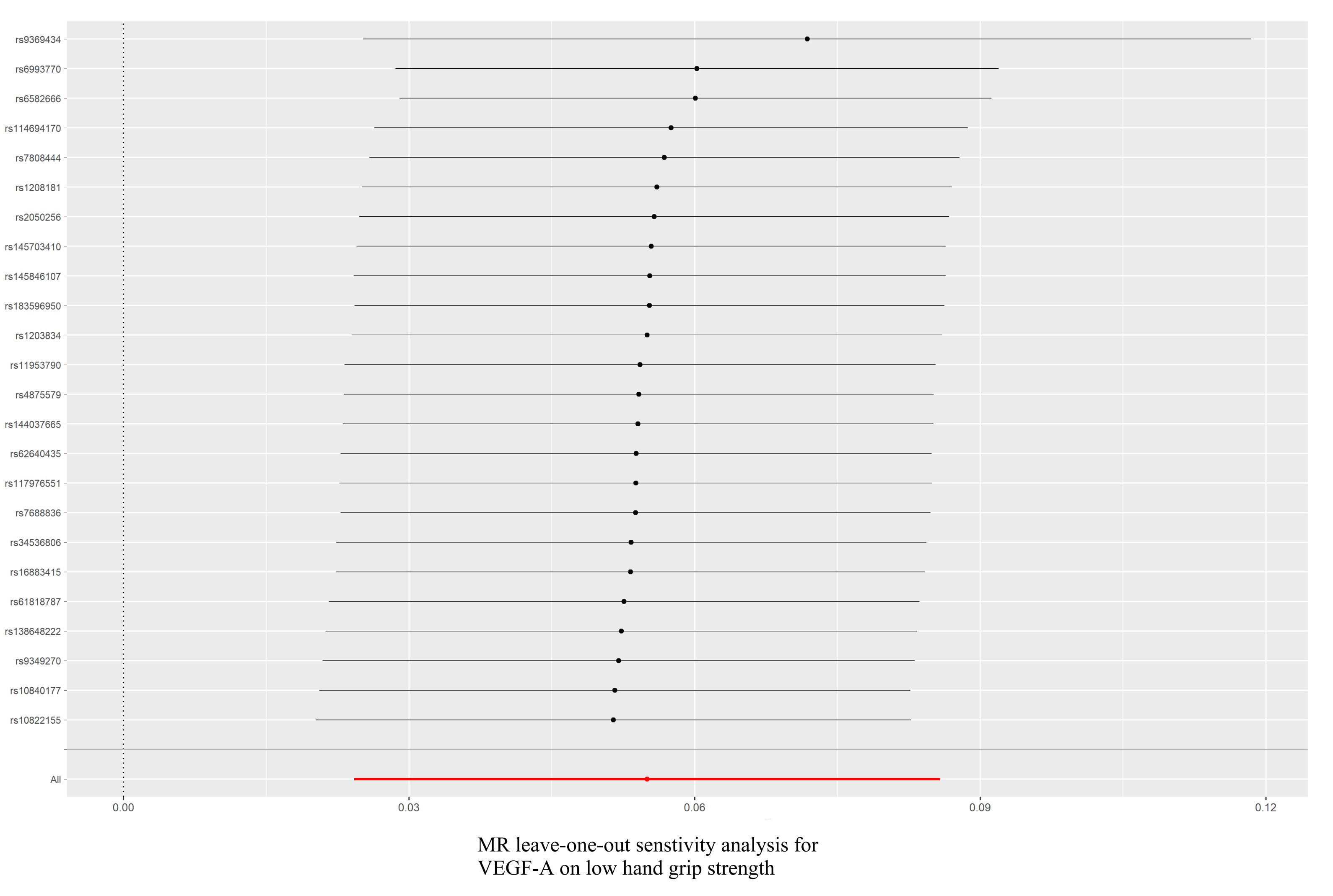

Supplement: Supplementary Figure 1 — MR leave-one-out senstivity analysis for VEGF-A on low hand grip strength. [file Image_1.JPEG]

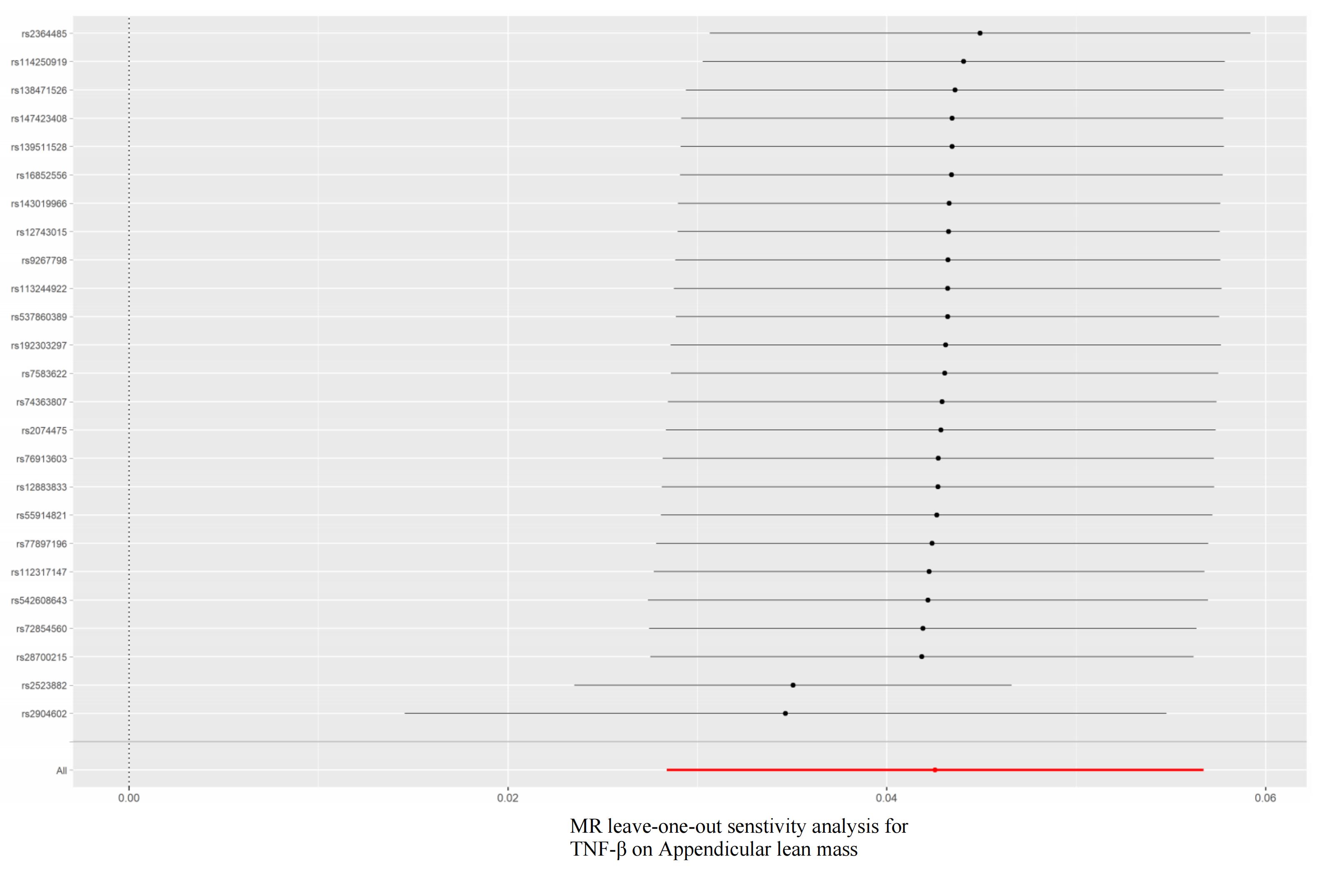

Supplement: Supplementary Figure 2 — MR leave-one-out senstivity analysis for TNF-β on appendicular lean mass. [file Image_2.JPEG]
